# Mechanisms of first-line antimicrobial resistance in multi-drug and extensively drug resistant strains of *Mycobacterium tuberculosis* in KwaZulu-Natal, South Africa

**DOI:** 10.1186/s12879-016-1906-3

**Published:** 2016-10-26

**Authors:** Navisha Dookie, A. Willem Sturm, Prashini Moodley

**Affiliations:** Medical Microbiology and Infection Prevention and Control, School of Laboratory Medicine and Medical Science, College of Health Science, University of KwaZulu-Natal, Durban, South Africa

**Keywords:** Drug-resistance, First-line drugs, Multi-drug resistant-TB, Extensively drug-resistant-TB, Clonality

## Abstract

**Background:**

In South Africa, drug resistant tuberculosis is a major public health crisis in the face of the colossal HIV pandemic.

**Methods:**

In an attempt to understand the distribution of drug resistance in our setting, we analysed the *rpoB*, *katG, inhA*, *pncA* and *embB* genes associated with resistance to key drugs used in the treatment of tuberculosis in clinical isolates of *Mycobacterium tuberculosis* in the KwaZulu-Natal province.

**Results:**

Classical mutations were detected in the *katG, inhA* and *embB* genes associated with resistance to isoniazid and ethambutol. Diverse mutations were recorded in the multidrug resistant (MDR) and extensively drug resistant (XDR) isolates for the *rpoB* and *pncA* gene associated with resistance to rifampicin and pyrazinamide.

**Conclusions:**

*M.tuberculosis* strains circulating in our setting display a combination of previously observed mutations, each mediating resistance to a different drug. The MDR and XDR TB isolates analysed in this study displayed classical mutations linked to INH and EMB resistance, whilst diverse mutations were linked to RIF and PZA resistance. The similarity of the XDR strains confirms reports of the clonality of the XDR epidemic. The successful dissemination of the drug resistant strains in the province underscores the need for rapid diagnostics to effectively diagnose drug resistance and guide treatment.

## Background

Tuberculosis (TB) remains one of the greatest public health concerns of our time, exacerbated by drug-resistant strains of *Mycobcterium tuberculosis* and co-infection with human immunodeficiency virus (HIV). In its latest global TB report in 2015, the world health organization (WHO) estimated that 3.5 % of new cases and 20.5 % previously treated patients have multi-drug resistant (MDR) TB; i.e. resistance to isoniazid (INH) and rifampicin (RIF). Of these cases, 9 % have extensively drug resistant (XDR) TB. XDR TB strains display resistance to INH, RIF and additional resistance to a fluoroquinolone antibiotic and one of the three injectable second line agents: amikacin (AMIK), kanamycin (KAN) and capreomycin (CAP) [1].

Despite the implementation of therapeutic regimes combining INH, RIF, ethambutol (EMB) and pyrazinamide (PZA) [2], the escalation of MDR strains has compromised the utility of this drug combination. The morbidity and mortality rates associated with drug resistant TB is several times higher than with drug susceptible forms [1]. Treatment of drug resistant TB is further complicated by the decreased efficacy and higher toxicity associated with the second line drugs as well as the inability to provide early diagnostic data to guide treatment [3]. Conventional drug susceptibility testing relies on mycobacterial culture methods, providing results after weeks or months. Despite remarkable strides made in advancing diagnosis of drug-resistant TB with molecular-based diagnostics, such as the GeneXpert MTB/RIF assay, this technology currently only detects reistance to RIF [4].

The most common genes associated with resistance to the first-line drugs in *M.tuberculosis* have been identified based on the mode of action of each of the drugs and their demonstrated association with drug resistance. These include *ropB* (RIF), *katG* and *inhA* (INH), *pncA* (PZA) and *embB* (EMB). The clinical relevance of these mutations have been amply discussed [5, 6]. The recent application of whole genome sequencing has provided in-dept insight on the development and evolution of drug resistance, including the complexity of resistance in *M.tuberculosis*. Interestingly, over a hundered genetic loci implicated in drug resistance. A recent study identified mutations in fourty genes linked to INH resistance, highlighting the dynamic evolution of resistance-conferring mutations under drug pressure. Furthermore, muations associated with drug resistance play varying roles, which include causative or compensatory mutations or may result in increased fitness [3, 7]. Cohen et al. demonstrated that *M.tuberculosis* isolates bearing the most prevalent RIF resistance mutation (*rpoB* S450L), were most likely to have to compensatory mechanisms to overcome fitness costs associated with the mutation [8]. In addition to this complexity, resistance-conferring mutations in *M.tuberculoisis* vary geographically and according to the strain type [3].

In the study described in this report, we analysed mutations in the *ropB*, *katG, inhA*, *pncA* and *embB* genes and their association to resistance to the key first-line antimicrobials in clinical isolates from the KwaZulu-Natal province of South Africa.

## Methods

### *M. tuberculosis* clinical isolates

*M. tuberculosis* clinical isolates were selected from the storage collection of the Infection Control laboratory, University of KwaZulu-Natal. The isolates were from sputum specimens obtained from patients presenting to the Church of Scotland Hospital in the Tugella Ferry region of KZN, South Africa from 2005 to 2009. At initial isolation, the drug susceptibility profiles of the isolates were established in our laboratory using the 1 % proportion method [9]. Sixty isolates were selected for the study: 10 drug susceptible (DS), 15 multi-drug resistant (MDR-TB) and 28 extensively drug resistant (XDR-TB). The H37Rv laboratory strain was included as a control.

### Determination of the Minimum Inhibitory Concentration (MIC)

MIC was established using a multipoint inoculation technique on Middlebrook 7H10 agar medium supplemented with oleic acid-albumin-dextrose-catalase (OADC). Test plates contained INH, RIF and EMB at concentrations of 0.125; 0.25; 0.5; 1; 2; 4; 8; 16; 32; 64 and 128 mg/L. The plates were seeded with an *M.tuberculosis* suspension matched to spectrophotometer absorbance reading of 1 at a wavelenght of 600 nm. Test plates were incubated at 37 °C for 21 days in the presence of 5 % CO_2_. The MIC of an isolate was recorded as the lowest antibiotic concentration that inhibited growth of the organism. Resistance to INH, RIF and EMB was defined as concentrations of 0.2, 1.0 and 5.0 mg/L respectively, in accordance to WHO guidelines [10]. Isolates were tested in triplicate to ensure test accuracy and reproducibility. Due to the technical difficulty associated with conducting PZA testing in an acid based medium, MIC’s for the drug was not conducted.

### Genomic DNA extraction & amplification

Genomic DNA was extracted from cultures grown on Middlebrook 7H11 media using the CTAB-NaCl (Cetyl-trimethyl-ammonium Bromide-Sodium Chloride) method, as described previously [11]. The integrity and concentration of the DNA was determined using a NanoDrop 2000c spectrophotometer (Thermo Scientific). PCR amplification assays were carried out for the *inhA, katG, rpoB, pncA* and *embB* genes. Primers for each of the genes were selected from published literature or designed using Primer3 design software [12]. The Expand Hi Fidelity PCR kit (Roche) was used in accordance to the guidelines set out by the manufacturer. Table 1 contains specific annealing temperatures and primer sequences used for amplification.

**Table 1:** Primer sequences and annealing temperatures used for PCR and sequencing

Gene	Primer	Nucleotide Sequence (5′ → 3′)	Annealing Temp (°C)	Associated Drug Resistance	Ref
*rpoB*	*rpoB* F	TGTTGGACATCTACCGCAAG	54 °C	Rifampicin	*
	*rpoB* R	CGAGACGTCCATGTAGTCCA			
*inhA*	*inhA* F	CTACATCGACACCGATATGAC	55 °C	Isoniazid	[26]
	*inhA* R	GACCGTCATCCAGTTGTAG			
*katG*	*katG* F	GGTCGACATTCGCGAGACGTT	57 °C	Isoniazid	[27]
	*katG* R	TTGTTCCTGCGACGCATCGTG			
*pncA*	*pncA* F	GCTGGTCATGTTCGCGATCG	59 °C	Pyrazinamide	[28]
	*pncA* R	GCTTTGCGGCGAGCGCTCCA			
*embB*	*embB* F	AAGCTGGCGCACCTTCAC	55 °C	Ethambutol	*
	*embB* R	ATAGCGCGGTGATCAAAAA			

* newly designed primers

### DNA sequencing & analysis

Prior to sequencing, the quality of PCR amplicons were determined on a 1 % agarose gel. Amplicons were purified using the Invitrogen PureLink PCR purification kit (Applied Biosystems) and sequenced using ABI Prism Big Dye Terminator cycle sequencing kit V3.1 (Applied Biosystems) together with the forward primers selected for PCR amplification. Nucleotide sequences were aligned to the H37Rv reference strain using Genious V5.5.7 (Biomatters) sequence analysis software [13].

### Genotyping

The genotypes of the isolates were established using the IS*6110* restriction fragment length polymorphism (RFLP) method, as described previously [14].

## Results

### RFLP analysis

RFLP analysis revealed that most of the DS isolates belonged to the Beijing family of strains. Three DS isolates had a unique profile and 1 isolate was a variant of the F11 strain family. The F28 strain family was the predominant genotype of the MDR isolates, whereas the remaining isolates belonged to the F15/LAM4/KZN (KZN) strain family. One MDR isolate recorded a unique profile and 1 was a variant of the F28 strain family. All of the XDR isolates analysed in the study belonged to the KZN strain family. Genotypes, mutations and associated phenotypes are shown in Tables 2, 3 and 4.

**Table 2:** Mutations in the *rpoB, katG, pncA* and *embB* genes with the associated MICs and genotypes of the drug susceptible isolates

Isolate	Genotype	MIC (mg/L)			Mutation Profile			
		RIF	INH	EMB	*rpoB*	*katG*	*pncA*	*embB*
TF1538	Unique	1	≤0.125	2	-	-	-	-
TF1413	Beijing	1	16	2	-	G1388T	-	-
TF1582	Beijing	1	≤0.125	2	-	G1388T	-	-
TF832	F11V	1	≤0.125	2	-	-	-	-
TF1519	Beijing	1	≤0.125	2	-	-	-	-
TF1001	Unique	1	≤0.125	2	-	-	-	-
TF933	Unique	1	≤0.125	2	-	-	-	-
P090811	Beijing	1	≤0.125	2	-	G1388T	-	-
P090802	Beijing	1	≤0.125	2	-	G1388T	-	-
P090804	Beijing	1	≤0.125	2	-	G1388T	-	-

**Table 3:** Mutations in the *rpoB, katG, pncA* and *embB* genes with the associated MICs and genotypes of the MDR isolates

Isolate	Genotype	MIC (mg/L)			Mutation Profile ^a^Associated amino acid substitutions			
		RIF	INH	EMB	*rpoB*	*katG*	*pncA*	*embB*
MODS11	KZN	8	8	8	T1355C	G944C	ΔC	A916G
MODS688	KZN	128	8	8	C1349T T1690C	G944C	T416G	A916G
TF44949	F28	32	16	8	C1349T	G944C	-	A916G
TF3251	KZN	128	16	16	G1303T C1360A	G944C	-	A916G
TF78838	F28	128	16	8	C1349T	G944C	T100G	A916G
TF2063	F28	128	16	16	C1349T	G944C	T100G	A916G
TF3203	F28	128	16	16	C1349T	G944C	T100G	A916G
TF1951	F28V	128	16	16	A1334T G1473C	G944C	-	A916G
TF64747	KZN	128	16	8	C1349T T1690C	G944C	T416G	A916G
TF2889	F28	64	16	8	C1349T T1355C	G944C	-	C1489A
TF2040	F28	128	16	16	C1349T	G944C	T100G	C1489A
MODS682	F28	128	16	16	-	G944C	-	-
TF36480	KZN	128	16	8	-	G944C	T100G	-
TF2034	Unique	64	16	16	C1349T T1355C	G944C A1343C	-	-
TF2153	F28	128	16	16	-	G944C	T100G	-

^a^ *rpoB:* C1303T = D435Y; A1334T = H445L; C1349T = S450L; T1355C = L452P; C1360A = P454T; G1473C = I491M; T1690C = Y564H

*katG:* G944C = S315T

*pncA:* ΔC = Insertion of cytosine at position 457; T100G = Y34D; T416G = L139G

*embB*: A916G = M306V; C1489A = Q506K

**Table 4:** Mutations in the *rpoB, katG, pncA* and *embB* genes with the associated MICs and genotypes of the XDR isolates

Isolate	Genotype	MIC (mg/L)			Mutation Profile ^a^Associated amino acid substitutions				
		RIF	INH	EMB	*rpoB*		*katG*	*pncA*	*embB*
TF1762	KZN	16	8	16	A1304G	T1355C	G944C	ΔC	A916G
MODS141	KZN	128	8	16	A1304G	T1355C	G944C	ΔC	A916G
MODS39	KZN	32	4	2	A1304G	T1355C	G944C	ΔC	A916G
MODS387	KZN	128	16	8	A1304G	T1355C	G944C	ΔC	A916G
MODS338	KZN	128	16	16	A1304G	T1355C	G944C	ΔC	A916G
MODS667	KZN	128	16	16	A1304G	T1355C	G944C	ΔC	A916G
MODS513	KZN	128	8	16	A1304G	T1355C	G944C	ΔC	A916G
MODS143	KZN	128	16	16	A1304G	T1355C	G944C	ΔC	A916G
TF1824	KZN	128	16	16	A1304G	T1355C	G944C	ΔC	A916G
TF1925	KZN	128	16	16	A1304G	T1355C	G944C	ΔC	A916G
TF66937	KZN	128	16	8	A1304G	T1355C	G944C	ΔC	A916G
TF80198	KZN	128	16	8	A1304G	T1355C	G944C	ΔC	A916G
TF80164	KZN	128	16	16	A1304G	T1355C	G944C	ΔC	A916G
TF1497	KZN	128	16	16	A1304G	T1355C	G944C	ΔC	A916G
TF75549	KZN	128	16	16	A1304G	T1355C	G944C	ΔC	A916G
TF31066	KZN	128	16	16	A1304G	T1355C	G944C	ΔC	A916G
TF739	KZN	128	16	16	A1304G	T1355C	G944C	ΔC	A916G
MODS370	KZN	128	16	16	A1304G	T1355C	G944C	ΔC	A916G
TF3181	KZN	128	16	16	A1304G	T1355C	G944C	ΔC	A916G
TF37806	KZN	128	16	16	A1304G	T1355C	G944C	ΔC	A916G
TF2981	KZN	128	16	16	A1304G	T1355C	G944C	ΔC	A916G
MODS334	KZN	128	16	16	-	-	G944C	ΔC	A916G
TF2038	KZN	128	16	16	A1304G	-	G944C	ΔC	A916G
TF3228	KZN	128	16	16	-	-	G944C	ΔC	A916G
TF25027	KZN	128	16	8	A1304G	-	G944C	ΔC	A916G
TF51648	KZN	128	16	16	A1304G	-	G944C	ΔC	A916G
TF49127	KZN	64	16	16	A1304G	-	G944C	ΔC	A916G
MODS 195	KZN	32	16	8	A1304G	-	G944C	ΔC	A916G

^a^ *rpoB:*A1304G = D435G; T1355C = L452P

*katG:* G944C = S315T

*pncA:* ΔC = Insertion of cytosine at position 457

*embB*: A916G = M306V; C1489A = Q506K

### *rpoB* mutations and RIF resistance

Majority (73 %) of the RIF resistant isolates had at least 1 mutation in the *rpoB* gene, while 5 RIF resistant (3 MDR, 2 XDR) isolates had no alteration within the *rpoB* gene. The *rpoB* mutations were of various types: (1) A → G substitution in codon 435 (A1304G, D435G); detected in 5 XDR isolates with an MIC range of 64–128 mg/L, belonging to the KZN family of strains (2). T → C substitution in codon 452 (T1355C, L452P); detected in 1 MDR isolate of the KZN family strain family with an MIC of 8 mg/L (3). C → T substitution in codon 450 (C1349T, S450L); detected in 6 MDR isolates which belonged to the KZN and F28 strain families with an MIC range of 32–128 mg/L. Double mutants included (4) C → T substitution in codon 435 (C1303T, D435Y) together with an C → A substitution in codon 454 (C1360A, P454T); detected in 1 MDR isolate of the KZN strain family with an MIC of 128 mg/L (5). A → T substitution in codon 445 (A1334T, H445L) and a G → C substitution in codon 491 (G1473C, I491M); detected in 1 MDR isolate, a variant of the F28 strain family with an MIC of 128 mg/L (6). The S450L and L452P detected in 2 MDR isolates of the KZN and F28 strain families with an MIC of 64 mg/L; (7) the D435G and L452P detected in 21 XDR isolates belonging to the KZN strain family with an MIC range of 32–128 mg/L (8). The S450L and a T → C substitution in codon 564 (T1690C, Y564H), detected in 2 MDR isolates of the KZN strain family with an MIC of 128 mg/L. No mutations were detected in any of the DS isolates screened.

### *inhA; katG* mutations and INH resistance

No mutations were detected in the *inhA* gene or its promoter region amongst the isolates screened in the study. The *katG* mutations were of 3 types (1). G → T substitution in codon 473 (G1388T; no amino acid alteration); detected in 5 DS isolates, all belonging to the Beijing family of strains (2). G → C substitution in codon 315 (G944C, S315T); detected in all of the MDR and XDR isolates studied. The MIC range of the isolates was 4–16 mg/L. The mutation was detected across all genotypes detected in the study (3). One MDR isolate, unique in its genotype had double mutations within the *katG* gene. In addition to the S315T mutation, an A → C substitution in codon 468 (A1343C, N468A) was detected, associated with MIC of 16 mg/L.

The remaining DS isolates had no alteration in the *katG* gene.

### *pncA* mutations and PZA resistance

*pncA* gene mutations were of 3 types (1). T → G substitution in codon 34 (T100G, Y34D); detected in 6 MDR isolates, 5 of which belonged to the F28 family and the remaining isolate belonged to the KZN family of strains (2). T → G substitution in codon 139 (T416G, L139G); detected in 2 MDR isolates, belonging to the KZN family of strains (3). Insertion of a cytosine at position 457, present in 1 MDR and all the XDR isolates screened, all belonging to the KZN strain family, resulting in a frameshift. The remaining MDR isolates and DS isolates had no alteration in the *pncA* gene.

### *embB* mutations and EMB resistance

The *embB* mutations were of 2 types (1). A → G substitution in codon 306 (A916G, M306V); detected in 9 MDR and all the XDR isolates, associated with an MIC range of 2–16 mg/L. The mutation was detected across all genotypes of the study (2). C → A substitution in codon 506 (C1489A, Q506K); detected in 2 MDR isolates, belonging to the F28 family of strains and associated with an MIC of 16 mg/L. The remaining MDR and DS isolates had no alteration in the *emB* gene.

Mutations of the *rpoB, inhA, katG, embB* and *pncA* genes, MIC’s and associated genotypes are shown in Tables 2, 3 and 4.

## Discussion

In this study, we report on the mechanisms mediating first-line drug resistance amongst clinical isolates from KwaZulu-Natal, South Africa. South Africa ranks amongst the top ten high burden countries of drug resistant tuberculosis worldwide. The overall incidence rate in the KwaZulu-Natal province alone currently exceeds the incidence rates for all types of TB in some low incidence countries, like the USA [15]. In an attempt to understand the molecular basis of first-line drug resistance in our setting, we sequenced the *ropB*, *inhA*, *katG, pncA* and *embB* genes associated with resistance to key drugs used in the treatment of DS tuberculosis.

Analysis of the *inhA*, *katG* and *embB* genes demonstrated classical mechanisms that have been associated with resistance to INH and EMB [5, 6]. No mutations were detected in the *inhA* gene or its promoter region in the isolates screened. This is in keeping with previous reports [16]. Common *inhA* mutations that have been reported occur in the *inhA* promoter region at position −15, its correlation, however, is strongest with INH mono-resistant isolates or isolates with low-level INH resistance [5, 6]. A recent study demonstrated that double mutations in the *inhA* gene, in the promoter and coding regions, resulted in high-level INH resistance [17]. A large number of RIF resistant isolates bear mutations in the *inhA* and its promoter region making these mutations high predictors of RIF resistance, despite being absent in a subset of INH resistant isolates.

The main mechanism mediating INH resistance in the isolates studied is the *katG* S315T mutation that was detected in all the MDR and XDR isolates. Numerous reports have found this mutation to be the most common mutation associated with INH resistance. One MDR isolate with unique genotype had double mutations in the *inhA* gene:the S315T mutation occurred in conjunction with the N468A mutation. The double mutant did not record a higher MIC as compared to the other isolates bearing the S315T mutation only. The N468A appears to be novel, but may represent a natural polymorphism or phylogenetic marker of the unique genotype of the isolate. The G1388T mutation detected in the 5 DS isolates is natural polymorphism associated with the Beijing genotype that has no bearing on resistance.

Approximately 96 % of RIF resistance is attributed to mutations contained in an 81 bp hot-spot region known as the RIF resistance determining region (RRDR) which encompasses codons 507–533 of the *rpoB* gene [5, 6]. Mechanisms of resistance for the MDR and XDR isolates varied in the case of RIF resistance, despite the MICs falling within a similar range. Various mutation types were described amongst the MDR isolates. The S450L (corresponding to codon 531 in *E.coli*) mutation was the most common, accounting for resistance in 7 MDR isolates, in keeping with various reports. Four MDR Isolates had the S450L mutation together with L452P (corresponding to codon 533 in *E.coli*) or the T564H mutation [16]. One MDR isolate had the L452P mutation only. One isolate had the H445L (corresponding to codon 526 in *E.coli*) and the I491M mutation [16]. To the best of our knowledge, the I491M and T564H mutations are novel and appear to be involved in mediating RIF resistance.

The main mutation mediating RIF resistance in the XDR isolates was the D435G mutation together with the L452P mutation. This was detected in all the XDR isolates and 1 MDR isolate. Three isolates had the D435G mutation only and one isolate had the S450L mutation. Although the mutations and genotypes of the isolates were diverse, all mutations correlated with high level RIF resistance. Interestingly, the MDR and XDR isolates both had mutations in codon 435 but each resulted in different amino acid substitutions. Similar findings were described for KZN MDR and XDR isolates in a study by Ioger et al. [16] analysing the whole genome sequences of drug resistant isolates KZN strain family. The main mechanism mediating resistance in the KZN MDR isolate was attributed to the D435Y mutation and the D435G and L452P mutations in the KZN XDR isolates. The study only analysed the KZN strain family [16]. Our study shows a greater diversity in the MDR RIF resistance mechanisms and the isolates represented various strain families. In our study, the mutation in codon 435 was responsible for resistance in most of the MDR isolates. In contrast, Ioger et al. reported that this mutation was only responsible for 9 % of RIF resistance [16].

Resistance to PZA, as in the case of RIF was represented by diverse mutations in the MDR and XDR isolates. The MDR isolates had a mutation either in codon 34 (nucleotide 100) or codon 139 nucleotide 416), while XDR isolates had an insertion of a cytosine at position 457, leading to a frameshift in the amino acid translation. The insertion was also detected in one MDR isolate, possibly with a higher level of resistance to PZA. Due to technical difficulty associated with PZA susceptibility testing, no MICs were carried out for the drug. Instead, we sought to identify mutations in the MDR and XDR groups and compare them with published literature. Mutations at position 100 have been described in isolates in Japan and Peru while mutations at position 416 have been reported South Africa, Thailand, China, USA, Portugal, Spain and Singapore [18–26]. The insertion at position 457 has been described in isolates from Brazil [27].

Mutations within the *pncA* gene are highly diverse, with 600 unique mutations at 400 different positions reported to date [28]. In keeping with this diversity, the study by Ioger et al. showed different mechanisms of PZA resistance in the MDR and XDR isolates from the KZN strain family as compared to the mechanisms described in this report [14]. This highlights the difficulty associated with detection of PZA resistance. The diversity of the mutations detected in the isolates varies greatly, making it impossible to apply to molecular diagnostic assay. PZA susceptibility testing poses a further challenge. The PZase enzyme required for the conversion of PZA to its active form is only functional at an acid pH, making it difficult to test the drug using conventional media. With a prevalence of approximately 60.5 % in patients with confirmed MDR TB, PZA resistance testing is of utmost importance as PZA forms an integral role in current multidrug regimens and is also a key component of new treatment regimens undergoing evaluation in phase II or III clinical trials [29, 30].

A few isolates resistant to RIF and PZA did not display any mutations in the related genes. This phenomena has been previously described [5, 6]. This suggests that alternate resistance mechanisms may exist that remain to be identified. The differences in resistance mechanisms in the MDR and XDR isolates suggest that the strains emerged separately and acquired resistance mutations independently. This is against the premise that the XDR phenotype had evolved directly from the MDR phenotype, consistent with previous reports [14].

## Conclusion

In conclusion, we demonstrate that the *M.tuberculosis* strains circulating in our setting display a combination of previously observed mutations, each mediating resistance to a different drug. The MDR and XDR TB isolates analysed in this study displayed classical mutations linked to INH and EMB resistance, whilst diverse mutations were linked to RIF and PZA resistance. The observation that all XDR-TB isolates appear identical (KZN strain) is consistent with reports endemic transmission of XDR-TB in our region. This strain is highly virulent and has displayed increased fitness despite the burden of carrying resistance conferring mutations [31]. The results of the study underscores the need for rapid drug susceptibility testing to improve treatment efficacy and subsequent treatment outcomes. Future studies are required to determine that factors that drive the dynamic evolution of *M.tuberculosis* drug resistance under antibiotic pressure in patients over time. Whilst various new developments offer a ray of hope, these are years away from integration into TB programmes. Thus strengthening of public health systems and the stringent antibiotic stewardship remain critical in TB control efforts.

## Abbreviations

AMIK: Amikacin
DS: Drug susceptible
EMB: Ethambuthol
HIV: Human immunodeficiency virus
INH: Isoniazid
KAN: Kanamycin
MDR: Multidrug resistant
OADC: Oleic acid albumin dextrose catalase
PZA: Pyrazinamide
RFLP: Restriction fragment length polymorphism analysis
RIF: Rifampicin
TB: Tuberculosis
WHO: World Health Organization
XDR: Extensively drug resistant
